# Comparison of Carbonic Anhydrases for CO_2_ Sequestration

**DOI:** 10.3390/ijms23020957

**Published:** 2022-01-16

**Authors:** Franziska Steger, Johanna Reich, Werner Fuchs, Simon K.-M. R. Rittmann, Georg M. Gübitz, Doris Ribitsch, Günther Bochmann

**Affiliations:** 1 Institute of Environmental Biotechnology, Department for Agrobiotechnology, University of Natural Resources and Life Sciences Vienna, Konrad Lorenz Str. 20, A-3430 Tulln, Austria; franziska.steger@boku.ac.at (F.S.); johanna.reich@boku.ac.at (J.R.); werner.fuchs@boku.ac.at (W.F.); guebitz@boku.ac.at (G.M.G.); guenther.bochmann@boku.ac.at (G.B.); 2 ACIB—Austrian Centre of Industrial Biotechnology, Krenngasse 37, 8010 Graz, Austria; 3 Archaea Physiology & Biotechnology Group, Department of Functional and Evolutionary Ecology, University of Vienna, Djerassiplatz 1, 1030 Vienna, Austria; simon.rittmann@univie.ac.at

**Keywords:** carbonic anhydrase, CO_2_ sequestration, thermostability, activity assay, recombinant expression

## Abstract

Strategies for depleting carbon dioxide (CO_2_) from flue gases are urgently needed and carbonic anhydrases (CAs) can contribute to solving this problem. They catalyze the hydration of CO_2_ in aqueous solutions and therefore capture the CO_2_. However, the harsh conditions due to varying process temperatures are limiting factors for the application of enzymes. The current study aims to examine four recombinantly produced CAs from different organisms, namely CAs from *Acetobacterium woodii* (AwCA or CynT), *Persephonella marina* (PmCA), *Methanobacterium thermoautotrophicum* (MtaCA or Cab) and *Sulphurihydrogenibium yellowstonense* (SspCA). The highest expression yields and activities were found for AwCA (1814 WAU mg^−1^ AwCA) and PmCA (1748 WAU mg^−1^ PmCA). AwCA was highly stable in a mesophilic temperature range, whereas PmCA proved to be exceptionally thermostable. Our results indicate the potential to utilize CAs from anaerobic microorganisms to develop CO_2_ sequestration applications.

## 1. Introduction

Due to the rapid growth of the world economy, there is a continual escalation in its carbon intensity. Carbon dioxide (CO_2_) emissions are rapidly increasing, making CO_2_ the most abundant greenhouse gas emitted by human activities [1,2,3]. Reducing the CO_2_ content in the atmosphere is imperative in meeting the UNFCCC climate goal of limiting the global temperature increase below 2 °C above pre-industrial levels [4]. Strategies for capturing CO_2_ from flue gasses are essential, until the ultimate goal of transition from non-renewable energy sources, such as natural gas and coal, to renewable energy sources is reached. Currently, there are various technologies and advances for capturing CO_2_ from flue gas, including chemical absorption of CO_2_ by solvents [5,6,7]. Through this method, a great fraction of CO_2_ can be depleted from the flue gas by passing it through a solvent, such as monoethanolamine (MEA), where CO_2_ molecules are extracted [8]. In the second step, the solvent is regenerated, and pure CO_2_ is released. A significant detrimental effect when utilizing MEA is the side generation of toxic wastes and aerosols [8,9]. Another disadvantage is the high amount of energy required for the release of CO_2_ from the solvent at high temperatures up to 120 °C [10], leaving CO_2_ capture with MEA unfavorable from an economic and sustainable standpoint [11].

An alternative is the “biomimetic” approach to capture CO_2_. Enzymes, such as carbonic anhydrases (CAs), accelerate the hydration of CO_2_, consequently facilitating the application of energy-efficient but kinetically-limited aqueous solvents, such as salt solutions (e.g., CaCl_2_, KOH) [8,12]. Biomimetic CO_2_ capture has previously been described, studied and implemented as a more sustainable and more economic option [12,13,14]. The temperature needed to release pure CO_2_ from aqueous salt solutions can be significantly reduced to 80 °C or less in biomimetic applications due to the decreased binding of CO_2_ compared to MEA solutions [15,16]. An existing critical challenge is to maintain the stability of the enzymes during varying temperatures from 30 °C to 80 °C in the process [15,17].

CAs are mainly zinc-containing metalloenzymes [18] and hydrate CO_2_ to hydrogen carbonate (HCO_3_^−^) according to the following two-step mechanism [19,20]:

EZnOH^−^ + CO_2_ ↔ EZn(OH^−^)CO_2_ ↔ EZNHCO_3_^−^ ↔ EZnH_2_O + HCO_3_^−^ (1)

EZnH_2_O ↔ H^+^EZnOH^−^ + B ↔ EZnOH^−^ + BH^+^ (2)

CAs are ubiquitously found in prokaryotes as well as in eukaryotes. There are eight distinct classes of carbonic anhydrases (α, β, γ, δ, ζ, η, θ, ι), which vary in their roles in different crucial physiological processes, amino acid sequences and 3-D tertiary structures [18,21]. CAs from extremophiles, existing at high temperatures, are a specific focus of interest, because they have been proven to be particularly active and thermostable [12,22]. The following three CAs from thermophilic organisms were selected based on literature research: PmCA originates from the bacterium *Persephonella marina* EX-H1, which exists in the deep-sea in an environment with excessive temperatures up to 133 °C and high pressures. Under laboratory conditions, *P. marina* grows in temperatures up to 80 °C [23]. SspCA is an enzyme from the bacterial strain *Sulphurihydrogenibium yellowstonense* YO3AOP1, which occurs in hot springs with a growth optimum at 70 °C and can grow autotrophically with CO_2_. PmCA and SspCA are α-type CAs and have been studied intensely with existing patents that have been published for their uses in biomimetic CO_2_ capture applications [24,25,26,27,28]. Both enzymes have been demonstrated to have high hydratase activities and to be exceptionally thermostable [24,29,30]. MtaCA, also referred to as Cab in the literature, was selected to extend the study with a β-type, thermostable CA [31], originating from the extreme thermophilic archaeon *Methanobacterium thermoautotrophicum,* which grows at up to 75 °C and metabolizes CO_2_ to methane [32]. The fourth CA selected for this study was AwCA, also referred to as CynT in the literature. It originates from the bacterium *Acetobacterium woodii* DSM 1030 with a growth optimum at 30 °C [33]. According to the NCBI’s Conserved Domain Database (CDD), AwCA belongs to the β-type class [34]. Despite AwCA originating from a mesophilic organism, it was selected due to its stated exceptional activity [35].

The recombinant expression of genes was the pivotal breakthrough allowing for the mass production of enzymes paving the way towards the industrial implementation of enzymes [36]. The current study aimed to examine these four recombinantly produced CAs from different organisms for biomimetic CO_2_ sequestration. Firstly, expression yields were determined using a single expression strategy in *Escherichia coli*. The effect of the CA addition on CO_2_ hydration at different temperatures was explored by applying the three CAs with the highest expression yields. A novel straightforward assay for the determination of CA activity was applied in this study, where measurement occurs close to industrial process conditions and temperatures [37]. The new assay allowed the characterization of CAs at temperatures exceeding 0 °C, which is unique concerning the commonly used Wilbur Anderson assay [38]. In the end, the thermostability of the two best performing CAs was compared, under the given conditions.

## 2. Results

### 2.1. Selection, Expression and Purification

**Figure 1:**
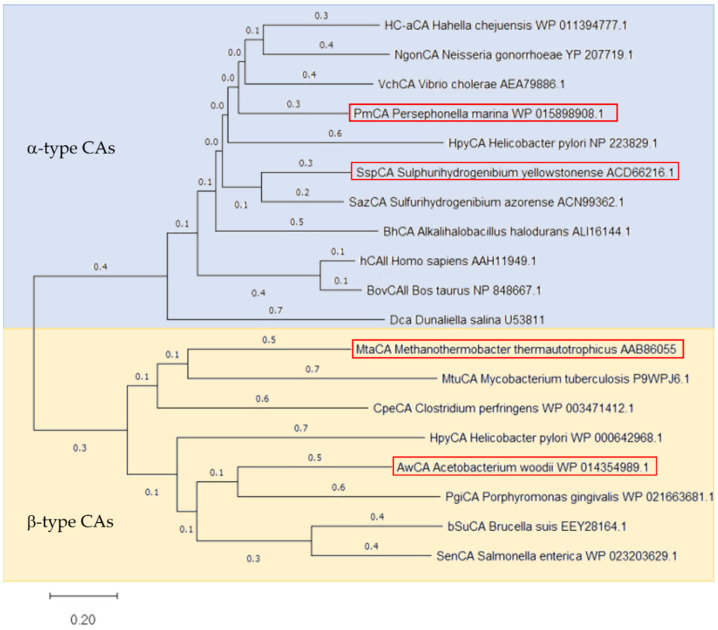
Phylogenetic tree of α-type and β-type CAs. Phylogenetic tree was constructed by MEGA11 software employing the Neighbor-joining Method [39] based on the protein sequences of CAs used in this study (highlighted by a red frame) as well as CAs described in literature. The blue background indicated α-type CAs, the yellow one the β-type CAs. The protein sequences are named by their Cryptonym followed by the microorganism’s name and the NCBI accession number. The bar at the bottom provides the scale of the branch lengths.

Four CAs were selected for comparison regarding their ability to convert CO_2_ into HCO_3_^−^, namely CAs from *Acetobacterium woodii* (AwCA), *Persephonella marina* (PmCA), *Methanobacterium thermoautotrophicum* (MtaCA) and *Sulphurihydrogenibium yellowstonense* (SspCA). The selected CAs belong to different types of CAs and share only low homologies among each other, as shown in the phylogenetic tree built on published CA protein sequences (Figure 1). PmCA and SspCA have been identified as α-type CAs originating from Gram-negative organisms. The two CAs share 45% homology and thus the highest similarity among the selected protein sequences. AwCA and MtaCA, however, are β-type CAs derived from Gram-positive organisms or archaea, respectively, and share only 20% homology among each other. Homologies between α-CAs and β-CAs are below 15%.

Both α- and β-CAs are metalloenzymes, which use Zn(II) ions for chemical catalysis. Despite their shared CO_2_ hydratase activity, the two classes demonstrate major structural differences, which are also seen in alignments of CAs investigated in this study (Supplementary Materials Section S3). The Zinc ion, for instance, is tetrahedrally coordinated by three histidines in α-CAs but by one histidine and two cysteines in β-CAs. A water molecule or hydroxide ion acts as a fourth ligand and nucleophile in the catalytic reaction in both CA classes. Similarly to all other α-CAs described in literature, the three histidines are present in PmCA (His89, His90, His108) and SspCA (His99, His101 and His118), which are relatively close to each other in the protein sequence. Additionally, a highly conserved histidine (His74 in PmCA and His64 in SspCA) is presently proposed to act as the proton shuttle. AwCA and MtaCA, however, are displaying the Zn(II) coordinating histidine (His110 and His87, respectively) and cysteines (Cys57 and Cys113 in AwCA, Cys32 and Cys90 in MtaCA) in their protein sequences, as expected for β-CAs. Besides, AwCA displays the highly conserved DSRV motif (residues 59–62) and Gln48, which are proposed to be involved in the catalytic reaction. Interestingly, Val62 is exchanged for Leu36 in this motif in MtaCA (residues 33–36) and also, Gln is not present at the corresponding amino acid position.

The protein sequences of PmCA and SspCA exhibit natural N-terminal signal peptides, comprising amino acids 1–19 in PmCA and 1-20 in SspCA, which were removed for the production of recombinant proteins in *E. coli*. Removal of the signal peptides of PmCA and SspCA was previously described as a tool to enhance expression yields [25,29,30]. The influence of the removal on hydratase activity was not evaluated during this study. Unlike the CAs mentioned above, AwCA and MtaCA do not reveal natural signal sequences. According to the analysis of the protein sequences, evidence for transmembrane domains or membrane anchors was not found in either of the applied CAs. According to Capasso and Supuran (2015), bacterial α-CAs are localized in the periplasmic compartment of Gram-negative bacteria. Contrastingly, bacterial β-CAs are mainly localized in the cytoplasm [40]. The alignment of sequences without signal peptides but fused to the StrepTag, as expressed in this study, can be found in the Supplementary Materials Section S3.

All CA encoding genes (Supplementary Materials Section S4) were codon optimized for expression in *E. coli* and fused to a C-terminal StrepTag, except for SspCA, which carried an N-terminal StrepTag. The synthetic genes were cloned into pET-vectors and expressed in *E. coli* BL21-Gold (DE3) at 37 °C. An SDS-PAGE analysis of samples withdrawn from *E. coli* liquid cultures at several time points (Supplementary Materials Figures S2, S5, S8 and S11) revealed high production levels of soluble protein at the expected molecular masses for AwCA (22.0 kDa), MtaCA (19.9 kDa) and PmCA (26.9 kDa), but only low production of SspCA (27.5 kDa). All CAs formed inclusion bodies, as determined by the SDS-PAGE analysis of insoluble cell fractions.

**Figure 2:**
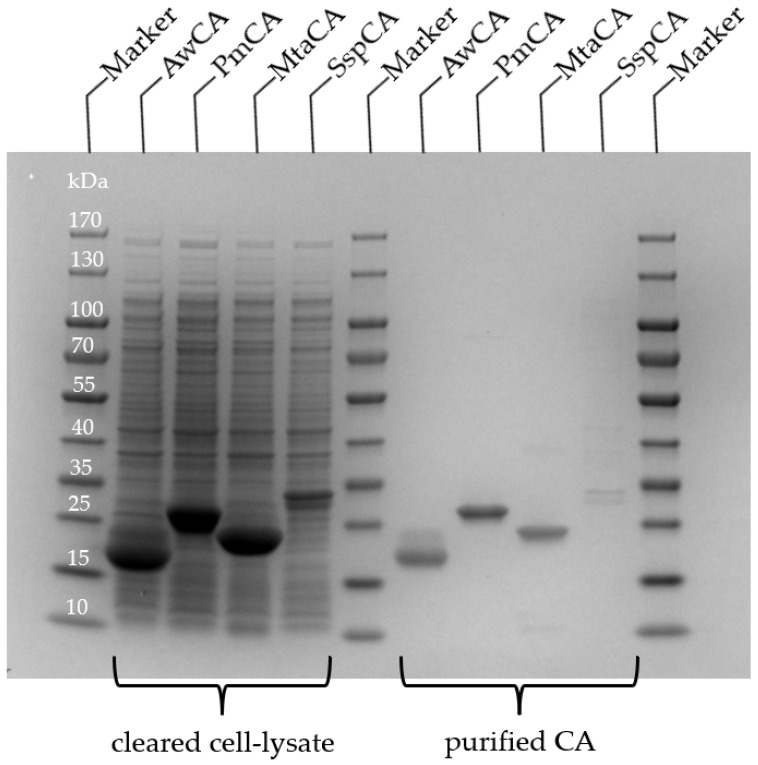
SDS-PAGE of CAs produced in *E. coli*. Samples of cleared cell lysate and after purification by affinity chromatography. Expected molecular masses are 22.0 kDa (AwCA), 26.9 kDa (PmCA), 19.9 kDa (MtCA) and 27.5 kDa (SspCA).

Purification of CAs from cleared *E. coli* cell lysates was achieved by a single step affinity chromatography through the StrepTag. Fractions containing the CAs were pooled and analyzed by SDS-PAGE (Figure 2). For SspCA, a double protein band on the SDS PAGE of cleared cell lysate and purified enzyme indicated a proteolysis on the N-terminal end during expression. The second protein band of cleared cell lysate and purified enzyme of AwCA in Figure 2 is attributed to the unspecific binding of *E. coli* proteins, whereas the second protein band in Supplementary Materials Figure S4 (elution fraction, line 7) is attributed to the formation of dimers of AwCA, which were not separated due to overloading of the SDS gel. AwCA belongs to the β-class, which is known to form dimers, tetramers and octamers [41], while α-CAs are usually monomers [40].

Typically, 25 mg AwCA, 15 mg PmCA, 18 mg MtaCA and 0.6 mg SspCA were obtained from 100 mL *E. coli* liquid culture according to the calculation based on purified enzymes. The yields are thus significantly higher than those from the literature, based on 100 mL liquid culture, which are 4.8 mg for AwCA when produced in *A. woodii* [35], and 0.129 mg or 0.93 mg of PmCA after production with and without signal peptide, respectively, in *E. coli* [29]. Around 10 mg MtaCA were obtained from diluted *E. coli* cell paste in 20 mL buffer [31]. In the study of Capasso et al. (2012), 12 mg SspCA were produced from an unknown volume of *E. coli* liquid culture using the expression vector pET15-b. Hence, the production of SspCA may be optimized in future studies.

SspCA was excluded from the following experiments after a change of an initial C-terminal StrepTag (not shown) to the N-terminal StrepTag resulted in similarly low expression yields. A further investigation to find the optimal expression system for SspCA was not conducted due to the fact that three out of four CAs demonstrated high expression yields under the chosen conditions. An optimization of the expression and vector system for SspCA may be conducted in future studies to assess the expression yields of SspCA.

### 2.2. Effect of CAs on CO_2_ Hydration at Different Temperatures

**Figure 3:**
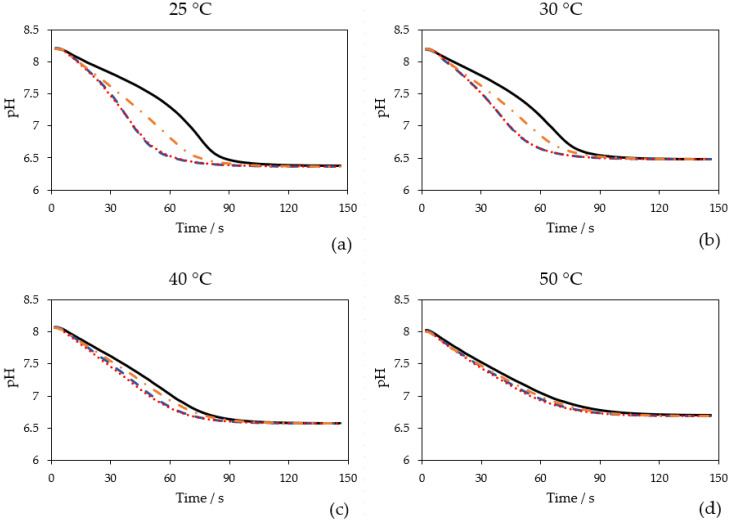
Time-dependent course of pH when 0.1 mol L^−1^ Tris-sulfate buffer was sparged with 100% CO_2_ (200 mL min^−1^) at different measurement temperatures: (**a**) 25 °C; (**b**) 30 °C; (**c**) 40 °C; (**d**) 50 °C. Without enzyme (black, solid line), with 0.25 mg L^−1^ AwCA (blue, dashed line), with 0.25 mg L^−1^ PmCA (red, dotted line), with 0.25 mg L^−1^ MtaCA (orange, dashed-dotted line). *n* = 3.

The effect on CO_2_ hydration at different temperatures (25 °C, 30 °C, 40 °C and 50 °C) was investigated for AwCA, PmCA and MtaCA. Figure 3 displays the pH courses over time when 0.1 mol L^−1^ Tris-sulfate buffer was sparged with CO_2_ at different temperatures. At each temperature, the blank without CA showed the slowest decrease in pH compared to the measurements where CAs were added. When CO_2_ is introduced into aqueous solutions it partly forms carbonic acid, which almost completely deprotonates into HCO_3_^−^ + H^+^, which causes a pH drop. CAs catalyze the direct conversion of CO_2_ to HCO_3_^−^ + H^+^ and vice versa [18]. Therefore, at a surplus of CO_2_, the more active the CA is, the faster the pH drops. The difference between the blank and CAs was most obvious at 25 °C to 40 °C, demonstrating that all enzymes had an accelerating effect on CO_2_ hydration in this temperature range. However, it is noticeable that the effect of MtaCA was constantly minor compared to PmCA and AwCA.

The advantage of the method used in this study is that the measurement is conducted at relevant temperatures of industrial processes (25–50 °C), in contrast to the widely used Wilbur Anderson assay, which is conducted solely at 0 °C [38]. Hence, the effect of CAs at temperatures close to process conditions can be investigated. Comparing the four diagrams (Figure 3), it is visible that with temperature elevation the equilibrium pH is rising because the solubility of CO_2_ is decreasing [42]. The starting pH at 40 °C and 50 °C was also lower being close to 8.0. The buffer stock solution (500 mL) was only preheated at the respective temperature before pH adjustment. For future measurements, it is recommended to keep the buffer stock solution in a temperature-controlled vessel during pH adjustment to avoid temperature-dependent pH shifts.

As illustrated in Figure 3, at higher temperatures the course of the blank becomes steeper and more similar to the curves in which CAs were added. At higher temperatures, the uncatalyzed formation of HCO_3_^−^ + H^+^ is faster than at lower temperatures. Whereas in total, less CO_2_ is solubilized, leading to a lower substrate concentration. At 50 °C, only a minor difference between the measurements with and without CAs can be observed. Another potential reason might be that the CAs are eventually less active at elevated temperatures. In this publication, the activity was calculated at 25 °C (Section 2.3). Calculation of activities at 30 °C, 40 °C and 50 °C was not performed. However, it might be unlikely that CAs that originate from thermophilic organisms (PmCA and MtaCA) lose their activity at 50 °C. As visualized in Figure 3, the effect of CA addition to CO_2_ capture at higher temperatures (>40 °C) is marginal and therefore not advisable. Fradette et al. (2017) reported that the absorption of CO_2_ takes place at 30–40 °C, while the desorption is conducted at 80 °C [15]. Hence, when CAs are recirculated together with the solvent, the thermostability of CAs is of higher interest than their activity at temperatures exceeding 40 °C.

The results for PmCA and MtaCA are in accordance with the literature where both enzymes are described to be thermophilic [29,31]. In prior studies, however, hydratase activity was only investigated at 0 °C (PmCA) [24,29,43] or room temperature (MtaCA) [31] utilizing the Wilbur Anderson assay [38] or an undescribed modification thereof [44], respectively. AwCA has not yet been described to be active at high temperatures. In contrast to PmCA and MtaCA [23,32], AwCA originates from a mesophilic organism [35], leading to the assumption that AwCA is only active at moderate temperatures around 30 °C. The results shown in Figure 3, however, indicate that AwCA has an accelerating effect on CO_2_ hydration at temperatures up to at least 40 °C. Notably, the enzymes were kept on ice before the measurements, so the effect at different temperatures cannot prove the thermostability of the enzymes for more than a few minutes; incidentally, this was the time one measurement required. The thermostability over a longer period was determined in the following experiments, which will be described in Section 2.4.

### 2.3. Calculated Hydratase Activities at 25 °C

To evaluate the different hydratase activities at 25 °C in commonly used units, turnover rate per mg enzyme and WAU per mg enzyme were calculated for AwCA, MtaCA and PmCA (Table 1). When comparing WAU per mg enzyme, it becomes visible that AwCA (1814 WAU mg^−1^) and PmCA (1748 WAU mg^−1^) were about three times more active than MtaCA (580 WAU mg^−1^). High hydratase activities are essential for the success of CO_2_ capturing applications. MtaCA was excluded from further investigations because of its lower measured and reported hydratase activities compared to AwCA and PmCA [31].

### 2.4. Thermostability of AwCA and PmCA

**Figure 4:**
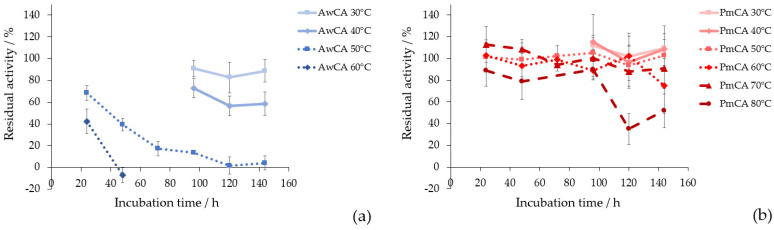
Residual hydratase activity over incubation time at different temperatures compared to unincubated enzyme for (**a**) AwCA at 30 °C, 40 °C and 50 °C; (**b**) PmCA at 50 °C, 60 °C, 70 °C, 80 °C. *n* = 3.

Thermostability is a crucial factor for the implementation of CAs in CO_2_ capture applications [15,17]. In Figure 4, residual hydratase activities after incubation at different temperatures over 24 h to 144 h are shown. The corresponding calculated activities can be found in Supplementary Materials Table S1. The Figure 4a shows the results for AwCA and the Figure 4b shows the results for PmCA. When AwCA was incubated at 30 °C for 144 h, 89 ± 10% of residual activity was retained. Incubation at 40 °C decreased residual activity to 59 ± 11% within 144 h. At 50 °C and 60 °C, residual activity drastically decreased to 4 ± 6% and −7 ± 7% after 144 h and 48 h of incubation, respectively. However, it is notable that AwCA retained 42 ± 11% residual after incubation at 60 °C for 24 h. Incubation at higher temperatures was not performed for AwCA due to the low stability at 60 °C compared to 30 °C and 40 °C (Figure 4). In contrast, PmCA turned out to be exceptionally thermostable, retaining 91 ± 19% residual activity after incubation at 70 °C for 144 h and 90 ± 14% residual activity after 96 h at 80 °C. When incubation time at 80 °C was prolonged to 144 h, PmCA still retained 52 ± 19% residual activity.

The thermostability of AwCA was tested in this study for the first time and therefore cannot be compared to the data in the literature. Regarding PmCA, the literature confirmed that this type of CA is fully active after incubation at 80 °C for 2 h [24]. Jo et al., 2014, tested the long-term stability of PmCA at 40 °C and 60 °C for 60 days, where PmCA retained 57% and 27% residual activity, respectively [43]. The current study further elucidates the insights on thermostability of PmCA, illustrating that significant amounts of activity were retained at 30 °C to 80 °C for up to 6 days (144 h). In summary, AwCA was stable long-term in a mesophilic temperature range and only short-term at temperatures exceeding 40 °C. In contrast, PmCA proved to be stable long-term at temperatures up to 80 °C.

## 3. Discussion

Thus far, α- and β-class CAs have been purified from various species [40,45]. Functional prokaryotic CAs have the advantage of being easily produced in *E. coli*, as in the cases of, e.g., β-class Caut-bCA from *Clostridium autoethanogenum* [46] and α-class HC-aCA from *Hahella chejuensis*. For the latter, expression yields (24.5 mg per 100 mL liquid culture) were similarly high as in the current study [47]. Contrastingly, bacterial α-class BhCA and β-class BCA were obtained from cell extracts of *Bacillus halodurans* and *Bacillus subtilis* SA3 cultures, respectively [48,49]. α-CA (Dca) from the green algae *Dunaliella salina* was functionally expressed in *E. coli* to a total amount of 15 mg. The culture volume was not given, but 1 g of wet *E. coli* cell pellet was reported to yield 1 mg of CA [50]. Marine species, such as mussels, diatoms and sea urchin exhibit functional CAs, which were mainly designated to the α-class [51,52,53,54]. In the literature, primarily their esterase activity rather than their hydratase activity was determined. CA of the marine diatom *Thalassiosira pseudonana* was successfully expressed in *E. coli* [53]. Expression yields were not reported. CAs from higher plants were mainly expressed in the original species for characterization, and hydratase activities were not reported [55,56]. For CO_2_ capture, prominent α-CAs from other eukaryotes were human hCAII, which was produced using bacterial vectors [13], and BovCAII, which was simply purified from bovine erythrocytes [57].

α- and β-class CAs differ in their structures, also demonstrated by the low homologies between the selected α- and β-CAs. Generally, the catalytic site of α-CAs was reported to be larger compared to β-CAs, thus resulting in higher hydratase activities [40]. When comparing the α- and β-CAs mentioned above, reported hydratase activities of α-class BhCA (3425 WAU mg^−1^) [48] and BovCAII (1749 WAU mg^−1^) [57] were higher compared to β-class BCA (1140 WAU mg^−1^) [49]. Accordingly, hydratase activities of α-class SspCA and hCAII were described to be amongst the highest with 7254 WAU mg^−1^ and 8000 WAU mg^−1^, respectively, in the literature [30,58]. However, there are exceptions, such as α-class HC-aCA (478 WAU mg^−1^) [47], Dca (400 WAU mg^−1^) [50] and CA from *T. pseudonana* (266 ± 49 WAU mg^−1^) [53], which exhibit comparably low hydratase activities. Although exhibiting very high hydratase activities (e.g., hCAII), the major drawback of eukaryotic α-CAs is that they are typically not thermostable [26].

In the current study, β-class AwCA (1814 WAU mg^−1^) demonstrated similarly high hydratase activity as the α-class PmCA (1748 WAU mg^−1^), whereas hydratase activity of the β-class MtaCA (580 WAU mg^−1^) was significantly lower. When comparing the protein structures, AwCA exhibited the typical features of β-CAs, whereas MtaCA varied by an exchanged Val62 for Leu36 in the highly conserved DSRV motif and missed Gln48. Both original amino acids are proposed to be involved in the catalytic reaction, potentially explaining the lower hydratase activity of MtaCA compared to AwCA. The highly conserved regions of α-CAs were found both in PmCA and SspCA. The lower hydratase activity of MtaCA compared to AwCA and PmCA during this study was in accordance with literature [31].

Kim et al. (2019) proposed that the enhanced thermostability of PmCA likely derives from the compactly folded homodimeric structure of α-CAs, which is stabilized by hydrophobic interactions, intramolecular disulfide bonds and interfacial networks of hydrogen bonds [59]. The current results confirm the exceptional thermostability of α-class PmCA compared to the mesophilic β-class AwCA. An exception might be the β-class MtaCA, which originates from a thermophilic organism and has been described to be stable after incubation for 15 min at up to 75 °C [31]; this is potentially explained by the closer relation to α-CAs in the phylogenetic tree (Figure 1). In the literature, SspCA was described as α-CA with one of the highest long-term thermostabilities while retaining its CO_2_ hydration activity after incubation at 100 °C for 3 h and 10% residual activity after incubation at 70 °C for 28 days [26,60]. However, due to the significantly lower expression yield of SspCA at the chosen conditions, the thermostability of SspCA was not evaluated in this study.

Consistently, CAs from thermophilic prokaryotes were already presented as the favorable candidates for biotechnological applications due to their enhanced thermostability in the literature, whereas CAs from other species are yet to be improved by protein engineering [12]. Immobilization may be an option to improve the thermostability of AwCA in future studies [61]. Production of SspCA may be increased significantly by using other expression vectors, as reported by Capasso et al. (2012) [30].

## 4. Materials and Methods

### 4.1. Chemicals and Reagents

All chemicals used in this work were of analytical grade. IPTG, ZnSO_4_, kanamycin, biotin and the buffer components were purchased from Sigma–Aldrich (St. Louis, MO, USA). Ampicillin and Nutrient Agar were purchased from Merck Millipore (Darmstadt, Germany). SDS buffer and the SDS-PAGE gels were purchased from Bio-Rad (Hercules, CA, USA). LB media was purchased from Carl Roth (Karlsruhe, Germany) and the protein marker for SDS PAGE from VWR International (Radnor, PA, USA). Bottled CO_2_ (purity 99.5%) was derived from Messer (Bad Soden, Germany).

### 4.2. Cloning, Expression and Purification

Genes coding for AwCA (accession number WP_014354989), PmCA (WP_015898908), MtaCA (AAB86055) and SspCA (ACD66216.1) were codon optimized and cloned over NdeI and HindIII restriction sites fused to a StrepTag for rapid purification by affinity chromatography. In the case of SspCA and MtaCA, the natural signal peptides were removed before cloning. Synthesis of genes and cloning into the expression vectors were provided by a commercial service (GenScript Biotech, Leiden, The Netherlands). Genes encoding AwCA, PmCA and MtaCA were cloned into vector pET26b(+) carrying a C-terminal StrepTag and the gene coding for SspCA into vector pET16b(+) providing a N-terminal StrepTag. The plasmids were transformed into *E. coli* BL21-Gold (DE3) (Agilent Technologies, Santa Clara, CA, USA). Freshly transformed *E. coli* cells were used to inoculate 20 mL LB-medium supplemented with 0.5 mmol L^−1^ ZnSO_4_ and 40 mg mL^−1^ Kanamycin for pET26b(+) or 100 µg mL^−1^ Ampicillin for pET16b(+) and cultivated overnight at 37 °C and 150 rpm. The overnight culture was used to inoculate 200 mL of the same medium in a 500 mL shake flask to an OD600 = 0.1. The culture was incubated at 37 °C and 150 rpm until an OD600 = 0.8 was reached. At this point, cells were induced by addition of IPTG to a final concentration of 0.1 mmol L^−1^ and incubated at 20 °C for 20 h (AwCA and PmCA) or 22 h (MtaCA and SspCA). Cultures were harvested by centrifugation at 4000 rpm and 4 °C for 30 min.

Enzymes were purified by affinity chromatography according to the manufacturer’s protocol (IBA GmbH, Goettingen, Germany). The elution fractions were collected, pooled and concentrated by vivaspin 20 columns (Sartorius, Göttingen, Germany). The buffer was exchanged by PD-10 desalting columns (GE Healthcare, Buckinghamshire, UK) with 0.1 mol L^−1^ Tris-HCl at a pH of 7. Enzymes were aliquoted to 500 µL and frozen at 20 °C until further usage.

### 4.3. CO_2_ Hydration Assay at Different Temperatures

Investigation of the effect of the CA addition on CO_2_ hydration at different temperatures was made by monitoring the pH change in buffered solution due to the formation of carbonic acid using the method as described by Fuchs et al., 2021 [37]. The reactions were conducted in a temperature-controlled vessel and the temperature was varied between 25–50 °C. Frozen enzyme stock solutions were thawed, diluted to 0.1 mg mL^−1^ with 0.1 mol L^−1^ Tris-HCl (pH 7) and kept on ice until the measurement. The reaction mix contained 40 mL reaction buffer (0.1 mol L^−1^ Tris-sulfate, pH 8.2) and 0.1 mL of the respective enzyme stock solution. The purified enzyme concentration in the reaction mix was 0.25 mg L^−1^, as determined by NanoPhotometer^®^ (NP80, IMPLEN, Westlake Village, CA, USA). For blank measurements, only the reaction buffer was measured. The pH of the reaction buffer was set to 8.2 at the respective measurement temperature before the experiments. CO_2_ gassing was started and the decline of pH during the experiment due to CO_2_ entry was recorded (Methrom Titrino, pH electrode: LL-Viscotrode and Software TiNet 2.5). Each measurement with or without CA was performed as a triplicate, and in the figures the mean values are shown.

### 4.4. Calculation of Hydratase Activities at 25 °C

Hydratase activities at 25 °C were calculated using the logged pH data. HCO_3_^−^ (mol L^−1^) was calculated according to the formula provided by Fuchs et al., 2021 [37]. Subsequently, HCO_3_^−^ was converted to µmol L^−1^ and depicted against time in a diagram. The slope of the graph between 7–18 s was taken as µmol CO_2_ converted to µmol HCO_3_^−^ L^−1^ s^−1^. The total turnover rate (µmol HCO_3_^−^ L^−1^ s^−1^) of the blank measurement was subtracted from the measurements with CAs before the calculation of the turnover rate per mg enzyme (µmol HCO_3_^−^ s^−1^ mg^−1^). To give the values in generally used units, a calibration curve with a standard enzyme (CA from bovine erythrocytes, Sigma–Aldrich, Taufkirchen, Germany) was used for conversion to Wilbur Anderson Units (WAU), as in Fuchs et al., 2021 [37].

### 4.5. Determination of Thermostability

Determination of thermostability was conducted using the same set-up as in Section 4.3. The measuring temperature was 25 °C. CA stock solutions were diluted to 0.1 mg mL^−1^ with 0.1 mol L^−1^ Tris-HCl at a pH of 7. Prior to the tests, aliquots of 1 mL were incubated at 30 °C, 40 °C, 50 °C, 60 °C, 70 °C or 80 °C in a heating block (Thermomixer comfort, Eppendorf, Hamburg, Germany) for up to 144 h. Prior to the CO_2_ hydration assay, the samples were cooled down to RT. For the calculation of the residual activity, the turnover rate per mg enzyme (µmol s^−1^ mg^−1^) was used.

## 5. Summary and Conclusions

Four CAs from different organisms were recombinantly produced in *E. coli*. Considering a later industrial application, the production and purification of the CAs were easy to perform and provided high yields using standard procedures. Under the chosen conditions, the expression yields of AwCA, PmCA and MtaCA were at least 25 times higher than for SspCA. Hence, SspCA was excluded from further experiments during this study. Nevertheless, a variation of the expression strategy for SspCA can potentially maximize the expression yield and should be part of future studies, as SspCA was reported to be one of the most active and thermostable CAs characterized so far in the literature.

A comparison of the effect on CO_2_ hydration at different temperatures, similar to industrial conditions, was performed for AwCA, PmCA and MtaCA using a novel assay. An accelerating effect on CO_2_ hydration was observed at 25 °C, 30 °C, 40 °C and up to 50 °C when enzymes were added to the buffer. However, there was only a minor difference between the CAs and the blank measurement at 50 °C, leading to the conclusion that the CA addition to CO_2_ capture at temperatures exceeding 40 °C is inapplicable. At 25 °C, hydratase activities were calculated for evaluation in general units. AwCA (1814 WAU mg^−1^) and PmCA (1748 WAU mg^−1^) were about three times more active than MtaCA (580 WAU mg^−1^), which led to the exclusion of MtaCA for the following thermostability experiments. AwCA was highly stable in a mesophilic temperature range with residual activities of 89 ± 10% and 59 ± 11% after incubation at 30 °C and 40 °C, respectively, for 144 h. PmCA was discovered to be exceptionally thermostable with residual activities of 91 ± 19% and 90 ± 14% after incubation at 70 °C for 144 h and at 80 °C for 96 h, respectively. The results of this study indicate that AwCA and PmCA might be suitable candidates for biomimetic CO_2_ sequestration at either mesophilic (AwCA and PmCA) or thermophilic (PmCA) temperature ranges. Further investigation of CAs from other species, especially those from thermophilic, anaerobic prokaryotes, is endorsed for broadening the range of possible candidates for CO_2_ sequestration.

## Figures and Tables

**Table 1:** Calculated hydratase activities at 25 °C for AwCA, MtaCA and PmCA in turnover rate per mg enzyme and WAU per mg enzyme.

Enzyme	Total Turnover Rate in µmol s^−1^ L^−1^	Turnover Rate Minus Blank in µmol s^−1^ L^−1^	Turnover Rate per mg Enzyme in µmol s^−1^ mg^−1^	WAU per mg Enzyme
Blank	793 ± 16	0	n. a.	n. a.
AwCA	1502 ± 45	710 ± 48	2839 ± 97	1814 ± 228
MtaCA	1204 ± 32	411 ± 36	1646 ± 73	580 ± 102
PmCA	1490 ± 13	698 ± 21	2790 ± 42	1748 ± 63

## Data Availability

The original data presented in this study are available in the Supplementary Materials and on request from the corresponding author.

## Supplementary Materials

The following are available online at https://www.mdpi.com/article/10.3390/ijms23020957/s1.

## References

[1] Canadell J.G., Le Quéré C., Raupach M.R., Field C.B., Buitenhuis E.T., Ciais P., Conway T.J., Gillett N.P., Houghton R.A., Marland G. (2007). Contributions to accelerating atmospheric CO_2_ growth from economic activity, carbon intensity, and efficiency of natural sinks. Proc. Natl. Acad. Sci. USA.

[2] Total Greenhous Gas Emission Trends and Projections in Europe. https://www.eea.europa.eu/data-and-maps/indicators/greenhouse-gas-emission-trends-7/assessment.

[3] Greenhouse Gas Emissions. https://www.epa.gov/ghgemissions/overview-greenhouse-gases.

[4] The Paris Agreement. https://unfccc.int/process-and-meetings/the-paris-agreement/what-is-the-paris-agreement.

[5] Benson S.M., Surles T. (2006). Carbon dioxide capture and storage: An overview with emphasis on capture and storage in deep geological formations. Proc. IEEE.

[6] Kanniche M., Gros-Bonnivard R., Jaud P., Valle-Marcos J., Amann J.-M., Bouallou C. (2010). Pre-combustion, post-combustion and oxy-combustion in thermal power plant for CO_2_ capture. Appl. Therm. Eng..

[7] Zhang Z., Wang T., Blunt M.J., Anthony E.J., Park A.-H.A., Hughes R.W., Webley P.A., Yan J. (2020). Advances in carbon capture, utilization and storage. Appl. Energy.

[8] Grant T., Anderson C., Hooper B. (2014). Comparative life cycle assessment of potassium carbonate and monoethanolamine solvents for CO_2_ capture from post combustion flue gases. Int. J. Greenh. Gas Control.

[9] Mazari S.A., Si Ali B., Jan B.M., Saeed I.M., Nizamuddin S. (2015). An overview of solvent management and emissions of amine-based CO_2_ capture technology. Int. J. Greenh. Gas Control.

[10] Kwak N.-S., Lee J.H., Lee I.Y., Jang K.R., Shim J.-G. (2012). A study of the CO_2_ capture pilot plant by amine absorption. Energy.

[11] Bhown A.S., Alto P. Direction of CO2 capture R&D. Proceedings of the NETL CO2 Capture Technology Meeting.

[12] Di Fiore A., Alterio V., Monti S.M., De Simone G., D’Ambrosio K. (2015). Thermostable carbonic anhydrases in biotechnological applications. Int. J. Mol. Sci..

[13] Bond G.M., Stringer J., Brandvold D.K., Simsek F.A., Medina M.-G., Egeland G. (2001). Development of integrated system for biomimetic CO_2_ sequestration using the enzyme carbonic anhydrase. Energy Fuels.

[14] González J.M., Fisher S.Z., Frost S.C., McKenna R. (2014). Carbonic anhydrases in industrial applications. Carbonic Anhydrase: Mechanism, Regulation, Links to Disease, and Industrial Applications.

[15] Fradette L., Lefebvre S., Carley J. (2017). Demonstration results of enzyme-accelerated CO_2_ capture. Energy Procedia.

[16] Savile C.K., Lalonde J.J. (2011). Biotechnology for the acceleration of carbon dioxide capture and sequestration. Curr. Opin. Biotechnol..

[17] Alterio V., Monti S.M., De Simone G., Frost S.C., McKenna R. (2014). Thermal-stable carbonic anhydrases: A structural overview. Carbonic Anhydrase: Mechanism, Regulation, Links to Disease, and Industrial Applications.

[18] Ozensoy Guler O., Capasso C., Supuran C.T. (2016). A magnificent enzyme superfamily: Carbonic anhydrases, their purification and characterization. J. Enzyme Inhib. Med. Chem..

[19] Boone C.D., Pinard M., McKenna R., Silverman D., Frost S.C., McKenna R. (2014). Catalytic mechanism of α-class carbonic anhydrases: CO_2_ hydration and proton transfer. Carbonic Anhydrase: Mechanism, Regulation, Links to Disease, and Industrial Applications.

[20] Lindskog S. (1997). Structure and mechanism of Carbonic Anhydrase. Pharmacol. Ther..

[21] Supuran C.T. (2016). Structure and function of carbonic anhydrases. Biochem. J..

[22] Littlechild J.A. (2015). Enzymes from extreme environments and their industrial applications. Front. Bioeng. Biotechnol..

[23] Götz D., Banta A., Beveridge T.J., Rushdi A.I., Simoneit B.R.T., Reysenbach A.-L. (2002). *Persephonella marina* gen. nov., sp. nov. and *Persephonella guaymasensis* sp. nov., two novel, thermophilic, hydrogen-oxidizing microaerophiles from deep-sea hydrothermal vents. Int. J. Syst. Evol. Microbiol..

[24] Borchert M.S. (2018). Heat-Stable Persephonella Carbonic Anhydrases and Their Use. U.S. Patent.

[25] Daigle R., Madore É., Fradette S. (2018). Techniques for CO2 Capture Using Sulfurihydrogenibium Sp. Carbonic Anhydrase. U.S. Patent.

[26] Di Fiore A., Capasso C., De Luca V., Monti S.M., Carginale V., Supuran C.T., Scozzafava A., Pedone C., Rossi M., De Simone G. (2013). X-ray structure of the first ‘extremo-α-carbonic anhydrase’, a dimeric enzyme from the thermophilic bacterium *Sulfurihydrogenibium yellowstonense* YO3AOP1. Acta Crystallogr. Sect. D Biol. Crystallogr..

[27] Rossi M. (2013). A New Heat-Stable Carbonic Anhydrase and Uses. Thereof. Patent.

[28] Vullo D., De Luca V., Scozzafava A., Carginale V., Rossi M., Supuran C.T., Capasso C. (2012). The first activation study of a bacterial carbonic anhydrase (CA). The thermostable α-CA from *Sulfurihydrogenibium yellowstonense* YO3AOP1 is highly activated by amino acids and amines. Bioorgan. Med. Chem. Lett..

[29] Kanth B.K., Jun S.-Y., Kumari S., Pack S.P. (2014). Highly thermostable carbonic anhydrase from *Persephonella marina* EX-H1: Its expression and characterization for CO_2_-sequestration applications. Process Biochem..

[30] Capasso C., De Luca V., Carginale V., Cannio R., Rossi M. (2012). Biochemical properties of a novel and highly thermostable bacterial α-carbonic anhydrase from *Sulfurihydrogenibium yellowstonense* YO3AOP1. J. Enzyme Inhib. Med. Chem..

[31] Smith K.S., Ferry J.G. (1999). A plant-type (β-class) carbonic anhydrase in the thermophilic methanoarchaeon *Methanobacterium thermoautotrophicum*. J. Bacteriol..

[32] Zeikus J.G., Wolfe R.S. (1972). *Methanobacterium thermoautotrophicus* sp. n., an anaerobic, autotrophic, extreme thermophile. J. Bacteriol..

[33] Balch W.E., Schoberth S., Tanner R.S., Wolfe R.S. (1977). *Acetobacterium*, a new genus of hydrogen-oxidizing, carbon dioxide-reducing, anaerobic bacteria. Int. Assoc. Microbiol. Soc..

[34] Marchler-Bauer A., Bo Y., Han L., He J., Lanczycki C.J., Lu S., Chitsaz F., Derbyshire M.K., Geer R.C., Gonzales N.R. (2017). CDD/SPARCLE: Functional classification of proteins via subfamily domain architectures. Nucleic Acids Res..

[35] Braus-Stromeyer S.A., Schnappauf G., Braus G.H., Gößner A.S., Drake H.L. (1997). Carbonic anhydrase in *Acetobacterium woodii* and other acetogenic bacteria. J. Bacteriol..

[36] Kirk O., Borchert T.V., Fuglsang C.C. (2002). Industrial enzyme applications. Curr. Opin. Biotechnol..

[37] Fuchs W., Steger F., Reich J., Ribitsch D., Rittmann S.K.M., Bochmann G. (2021). A simple and straightforward method for activity measurement of carbonic anhydrases. Catalysts.

[38] Wilbur K.M., Anderson N.G. (1948). Electrometric and colorimetric determination of carbonic anhydrase. J. Biol. Chem..

[39] Tamura K., Stecher G., Kumar S. (2021). MEGA11: Molecular Evolutionary Genetics Analysis Version 11. Mol. Biol. Evol..

[40] Capasso C., Supuran C.T. (2015). An overview of the alpha-, beta- and gamma-carbonic anhydrases from bacteria: Can bacterial carbonic anhydrases shed new light on evolution of bacteria?. J. Enzyme Inhib. Med. Chem..

[41] Rowlett R.S., Frost S.C., McKenna R. (2014). Structure and catalytic mechanism of β-carbonic anhydrases. Carbonic Anhydrase: Mechanism, Regulation, Links to Disease, and Industrial Applications.

[42] Dodds W.S., Stutzman L.F., Sollami B.J. (1956). Carbon dioxide solubility in water. Ind. Eng. Chem. Chem. Eng. Data Ser..

[43] Jo B.H., Seo J.H., Cha H.J. (2014). Bacterial extremo-α-carbonic anhydrases from deep-sea hydrothermal vents as potential biocatalysts for CO_2_ sequestration. J. Mol. Catal. B Enzym..

[44] Smith K.S., Jakubzick C., Whittam T.S., Ferry J.G. (1999). Carbonic anhydrase is an ancient enzyme widespread in prokaryotes. Proc. Natl. Acad. Sci. USA.

[45] Supuran C.T. (2008). Carbonic anhydrases—An overview. Curr. Pharm. Des..

[46] Pander B., Harris G., Scott D.J., Winzer K., Köpke M., Simpson S.D., Minton N.P., Henstra A.M. (2019). The carbonic anhydrase of *Clostridium autoethanogenum* represents a new subclass of β-carbonic anhydrases. Appl. Microbiol. Biotechnol..

[47] Ki M.R., Min K., Kanth B.K., Lee J., Pack S.P. (2013). Expression, reconstruction and characterization of codon-optimized carbonic anhydrase from *Hahella chejuensis* for CO_2_ sequestration application. Bioprocess Biosyst. Eng..

[48] Faridi S., Satyanarayana T. (2016). Novel alkalistable α-carbonic anhydrase from the polyextremophilic bacterium *Bacillus halodurans*: Characteristics and applicability in flue gas CO_2_ sequestration. Environ. Sci. Pollut. Res..

[49] Ramanan R., Kannan K., Vinayagamoorthy N., Ramkumar K.M., Sivanesan S.D., Chakrabarti T. (2009). Purification and characterization of a novel plant-type carbonic anhydrase from *Bacillus subtilis*. Biotechnol. Bioprocess Eng..

[50] Premkumar L., Bageshwar U.K., Gokhman I., Zamir A., Sussman J.L. (2003). An unusual halotolerant α-type carbonic anhydrase from the alga *Dunaliella salina* functionally expressed in *Escherichia coli*. Protein Expr. Purif..

[51] Pavičić-Hamer D., Baričević A., Gerdol M., Hamer B. (2015). *Mytilus galloprovincialis* carbonic anhydrase II: Activity and cDNA sequence analysis. Key Eng. Mater..

[52] Cardoso J.C.R., Ferreira V., Zhang X., Anjos L., Félix R.C., Batista F.M., Power D.M. (2019). Evolution and diversity of alpha-carbonic anhydrases in the mantle of the Mediterranean mussel (*Mytilus galloprovincialis*). Sci. Rep..

[53] Jensen E.L., Clement R., Kosta A., Maberly S.C., Gontero B. (2019). A new widespread subclass of carbonic anhydrase in marine phytoplankton. ISME J..

[54] Karakostis K., Costa C., Zito F., Brümmer F., Matranga V. (2016). Characterization of an alpha type carbonic anhydrase from *Paracentrotus lividus* sea urchin embryos. Mar. Biotechnol..

[55] Wang L., Liang J., Zhou Y., Tian T., Zhang B., Duanmu D. (2021). Molecular characterization of carbonic anhydrase genes in *Lotus japonicus* and their potential roles in symbiotic nitrogen fixation. Int. J. Mol. Sci..

[56] Chatterjee J., Coe R.A., Acebron K., Thakur V., Yennamalli R.M., Danila F., Lin H.-C., Balahadia C.P., Bagunu E., Padhma P.P.O.S. (2021). A low CO_2_-responsive mutant of *Setaria viridis* reveals that reduced carbonic anhydrase limits C_4_ photosynthesis. J. Exp. Bot..

[57] Da Costa Ores J., Sala L., Cerveira G.P., Kalil S.J. (2012). Purification of carbonic anhydrase from bovine erythrocytes and its application in the enzymic capture of carbon dioxide. Chemosphere.

[58] Ekinci D., Beydemir Ş., Alim Z. (2007). Some drugs inhibit in vitro hydratase and esterase activities of human carbonic anhydrase-I and II. Pharmacol. Reports.

[59] Kim S., Sung J., Yeon J., Choi S.H., Jin M.S. (2019). Crystal structure of a highly thermostable α-carbonic anhydrase from *Persephonella marina* EX-H1. Mol. Cells.

[60] Russo M.E., Olivieri G., Capasso C., De Luca V., Marzocchella A., Salatino P., Rossi M. (2013). Kinetic study of a novel thermo-stable α-carbonic anhydrase for biomimetic CO_2_ capture. Enzyme Microb. Technol..

[61] Sharma A., Bhattacharya A., Shrivastava A. (2011). Biomimetic CO_2_ sequestration using purified carbonic anhydrase from indigenous bacterial strains immobilized on biopolymeric materials. Enzyme Microb. Technol..

