# Supplementary material for: Comparison of Carbonic Anhydrases for CO2 Sequestration
Source: Int J Mol Sci. 2022 Jan 16;23(2):957. doi: 10.3390/ijms23020957 (PMC8777876; doi:10.3390/ijms23020957)
Supplement: Supplementary file 1 [file ijms-23-00957-s001.zip › ijms-1494492-supplementary.pdf]

International Journal of *Molecular Sciences*

MDPI

Article

Comparison of Carbonic Anhydrases for CO_2 Sequestration

Franziska Steger [1], Johanna Reich [1,2], Werner Fuchs [1], Simon K.-M. R. Rittmann [3], Georg M. Gübitz [1], Doris Ribitsch [2,*] and Günther Bochmann [1]

[1] Institute of Environmental Biotechnology, Department for Agrobiotechnology, University of Natural Resources and Life Sciences Vienna, Konrad Lorenz Str. 20, A-3430 Tulln, Austria; franziska.steger@boku.ac.at (F.S.); johanna.reich@boku.ac.at (J.R.); werner.fuchs@boku.ac.at (W.F.); guebitz@boku.ac.at (G.M.G.); guenther.bochmann@boku.ac.at (G.B.)

[2] ACIB —Austrian Centre of Industrial Biotechnology, Krenngasse 37, 8010 Graz, Austria

[3] Archaea Physiology & Biotechnology Group, Department of Functional and Evolutionary Ecology, University of Vienna, Djerassiplatz 1, 1030 Vienna, Austria; simon.rittmann@univie.ac.at

* Correspondence: doris.ribitsch@boku.ac.at

1. Original SDS-PAGE of CAs in Cleared Cell Lysate and After Purification

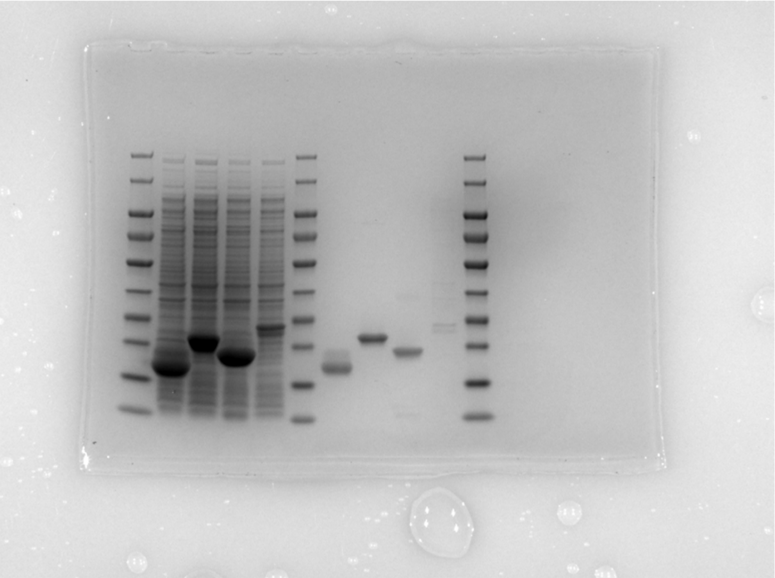

Figure S1. original SDS-PAGE of CAs expressed in *E. coli*. Samples of cleared cell lysate and after purification by affinity chromatography.

2. SDS-PAGEs of Timepoints During Expression and Purification of CAs

2.1. AwCA

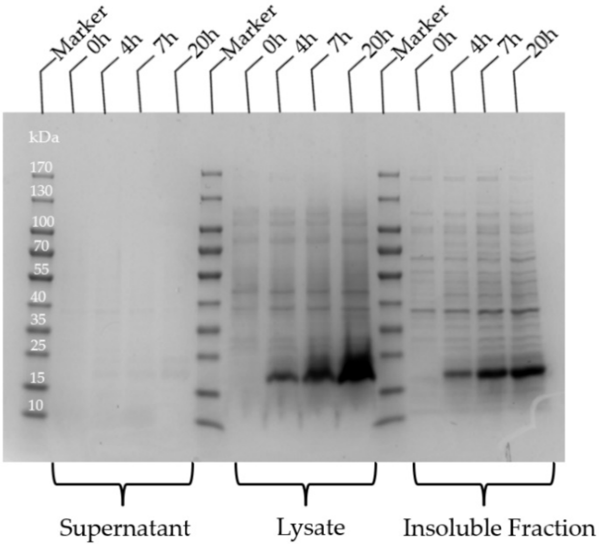

Figure S2. SDS-PAGE of supernatant, lysate and insoluble fraction during expression of AwCA in *E. coli*. Expected molecular weight is 22.0 kDa (AwCA).

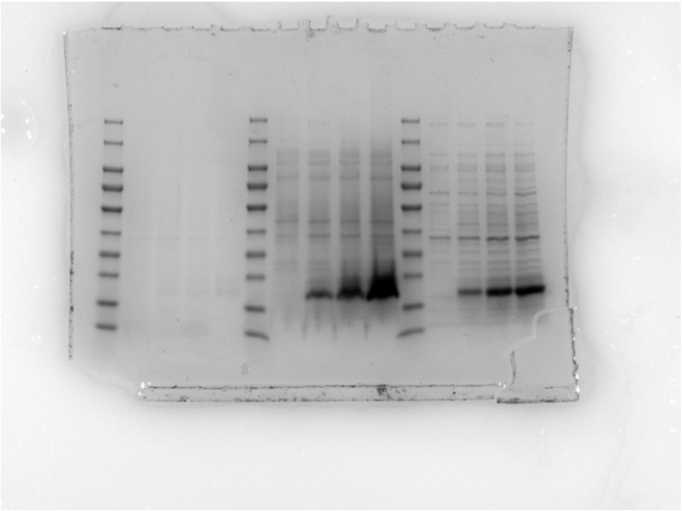

Figure S3. original SDS-PAGE of supernatant, lysate and insoluble fraction during expression of AwCA in *E. coli*.

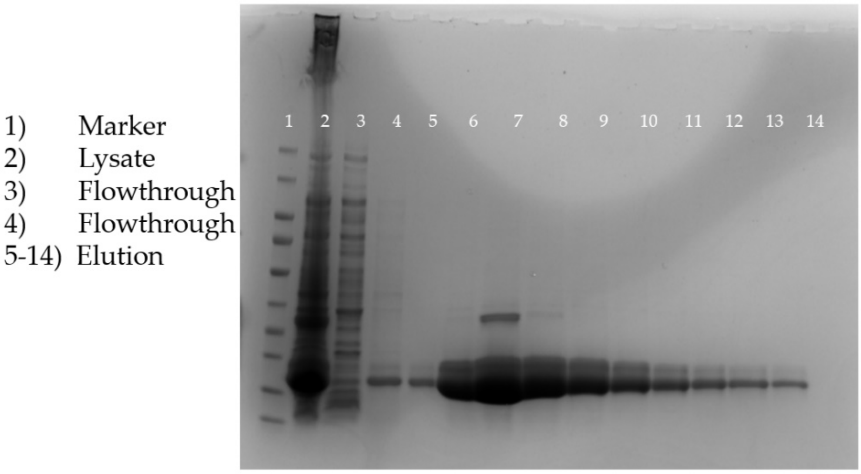

Figure S4. SDS-PAGE of lysate (2), flowthrough (3&4) and elution fractions (5-14) during purification of AwCA by affinity chromatography.

2.2. PmCA

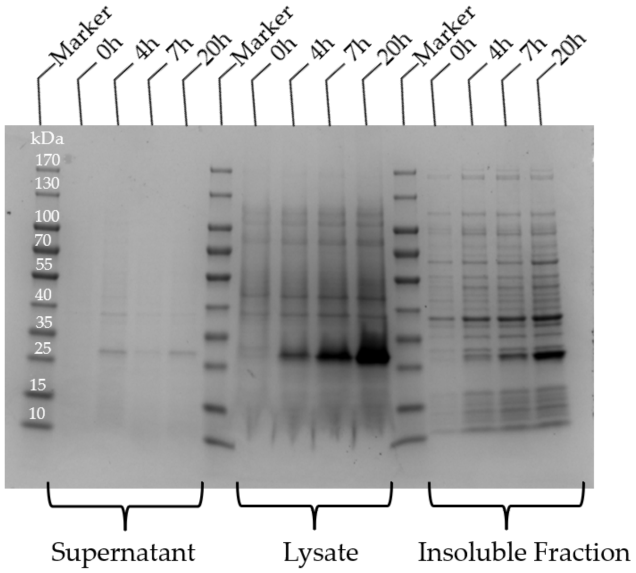

Figure S5. SDS-PAGE of supernatant, lysate and insoluble fraction during expression of PmCA in *E. coli*. Expected molecular weight is 26.9 kDa (PmCA).

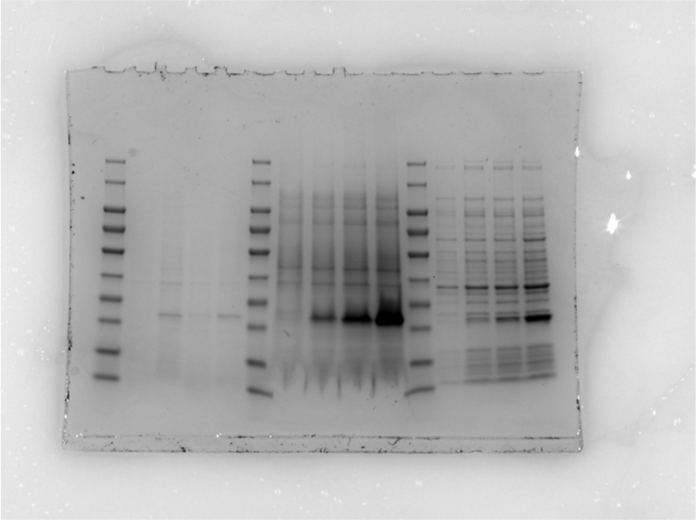

Figure S6. original SDS-PAGE of supernatant, lysate and insoluble fraction during expression of PmCA in *E. coli*.

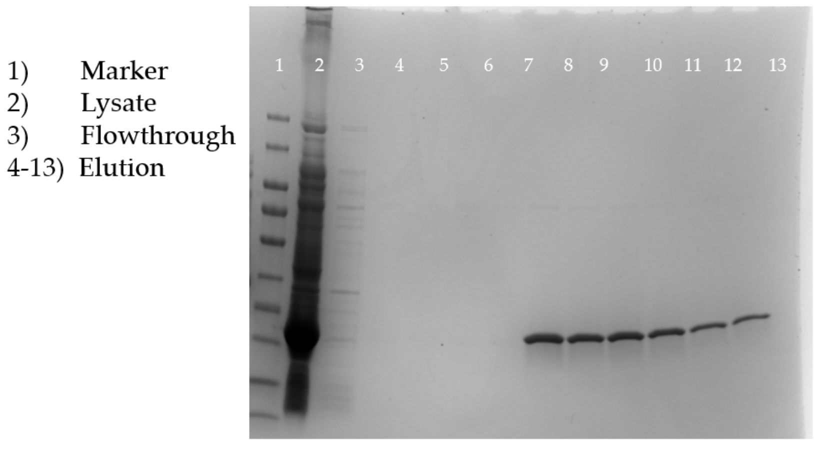

Figure S7. SDS-PAGE of lysate (2), flow through (3) and elution fractions (4-13) during purification of PmCA by affinity chromatography.

2.3. *MtaCA*

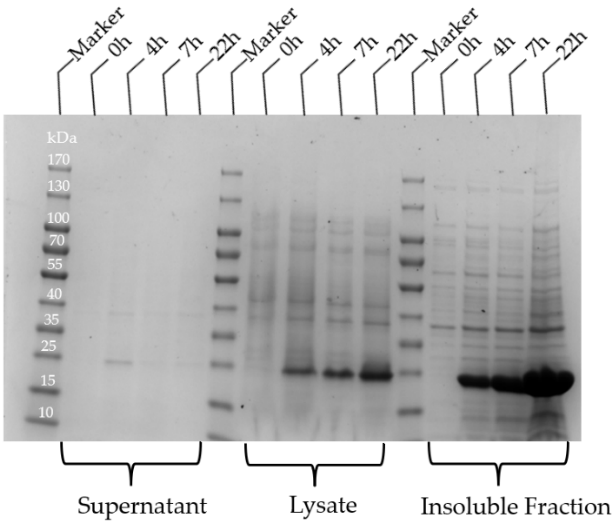

Figure S8. SDS-PAGE of supernatant, lysate and insoluble fraction during expression of MtaCA in *E. coli*. Expected molecular weight is 19.9 kDa (MtCA).

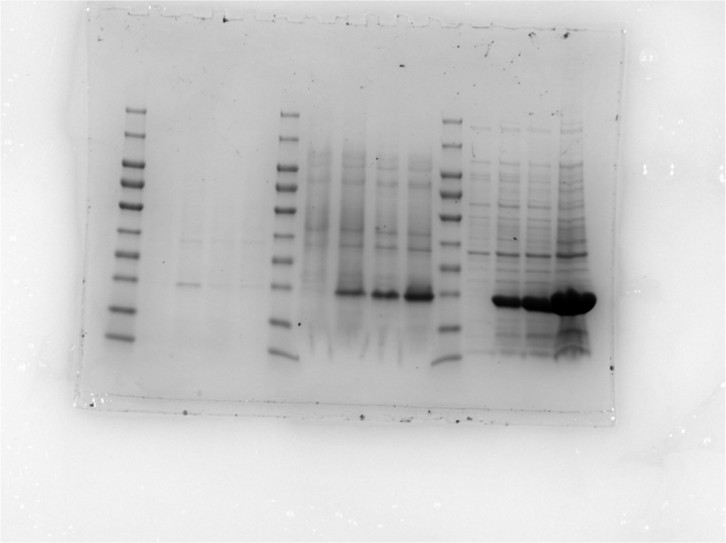

Figure S9. original SDS-PAGE of supernatant, lysate and insoluble fraction during expression of MtaCA in *E. coli*.

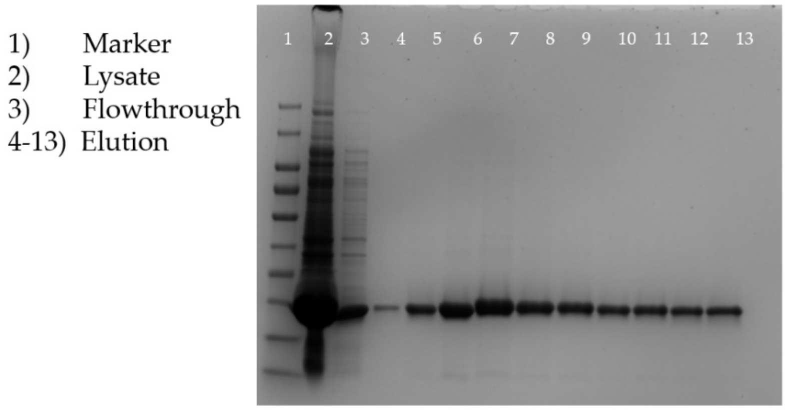

Figure S10. SDS-PAGE of lysate (2), flow through (3) and elution fractions (4-13) during purification of MtaCA by affinity chromatography.

2.4. SspCA

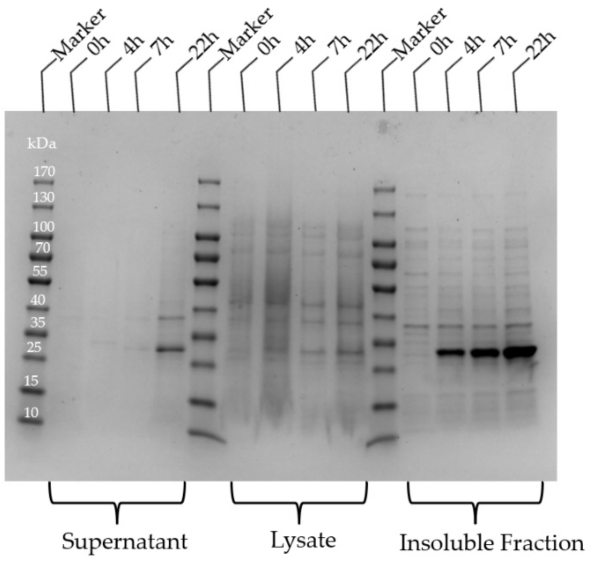

Figure S11. SDS-PAGE of supernatant, lysate and insoluble fraction during expression of SspCA in *E. coli*. Expected molecular weight is 27.5 kDa (SspCA).

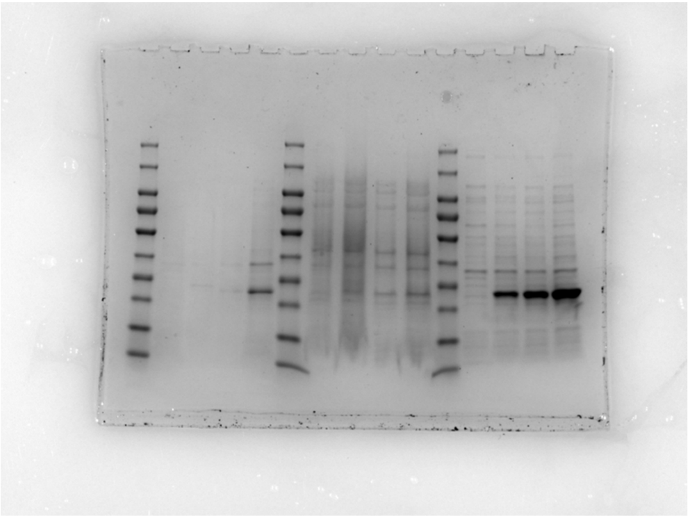

Figure S12. original SDS-PAGE of supernatant, lysate and insoluble fraction during expression of SspCA in *E. coli*.

1) Marker
2) Lysate
3) Flowthrough
4-11) Elution

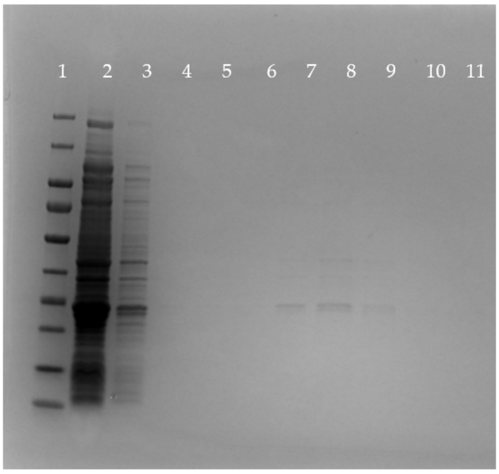

Figure S13. SDS-PAGE of lysate (2), flow through (3) and elution fractions (4-11) during purification of SspCA by affinity chromatography.

3. Protein Sequence Alignment of CAs as Expressed in this study

```
           ....|....| ....|....| ....|....| ....|....| ....|....|
                   10         20         30         40         50
PmCA       --------MG GGWSYHGEHG PEHWGDLKDE YIMCKIGKNQ SPVDINR--I
SspCA      MWSHPQFEKE HEWSYEGEKG PEHWAQLKPE FFWCKL-KNQ SPINIDKKYK
Consensus               WSY GE G PEHW  LK E     CK  KNQ SP   I

           ....|....| ....|....| ....|....| ....|....| ....|....|
                   60         70         80         90        100
PmCA       VDAKLKPIKI EYRAG-ATKV LNNGHTIKVS YEPGSYIVVD GIKFELKQFH
SspCA      VKANLPKLNL YYKTAKESEV VNNGHTIQIN IKEDNTLNYL GEKYQLKQFH
Consensus  V A L        Y        V  NNGHTI                G K  LKQFH

           ....|....| ....|....| ....|....| ....|....| ....|....|
                  110        120        130        140        150
PmCA       FHAPSEHKLK GQHYPFEAHF VHADKHGNLA VIGVFFKEGR ENPILEKIWK
SspCA      FHTPSEHTIE KKSYPLEIHF VHKTEDGKIL VVGVMAKLGK TNKELDKILN
Consensus  FH  PSEH         YP E HF VH     G     V GV   K G    N  L KI

           ....|....| ....|....| ....|....| ....|....| ....|....|
                  160        170        180        190        200
PmCA       VMPENAGEEV KLAHKINAED LLPKDRDYYR YSGSLTTPPC SEGVRWIVME
SspCA      VAPAEEGEKI -LDKNLNLNN LIPKDKRYMT YSGSLTTPPC TEGVRWIVLK
Consensus  V P   GE     L     N     L PKD   Y    YSGSLTTPPC  EGVRWIV

           ....|....| ....|....| ....|....| ....|....| ....
                  210        220        230        240
PmCA       EEMEMSKEQI EKFRKIMGGD TNRPVQPLNA RMIMEKWSHP QFEK
SspCA      KPISISKQQL EKLKSVMVNP NNRPVQEINS RWIIEGF--- ----
Consensus       SK Q  EK     M      NRPVQ   N  R I E
```

Figure S14: ClustalW Multiple Alignment (BioEdit Sequence Aligment Editor v7.0.5) of α-CAs from *Persephonella marina* (PmCA) and *Sulphurihydrogenibium yellowstonense* (SspCA) as expressed in this study. Orange: StrepTag. Grey background highlights the zinc-coordinating histidines, red background indicates the proton shuttle residues and blue background shows the „gate-keeper" residues of α-CAs.

```
             ....|....| ....|....| ....|....| ....|....| ....|....|
                     10         20         30         40         50
AwCA         MMMSEKKENV INGVIKKLKQ GNADFVKNEK NEGDISQMIR TKTVAEGQKP
MtaCA        ---------- --MIIKDILR ENQDFRFR-- ---DLSDLKH S--------P
Consensus                    IK       N DF          D S          P

             ....|....| ....|....| ....|....| ....|....| ....|....|
                     60         70         80         90        100
AwCA         YAVVITCSDS RVP--PEHIF SAGIGELFVI RTAGNVIGDF ELGSIEYAVG
MtaCA        KLCIITCMDS RLIDLLERAL GIGRGDAKVI KNAGNIVDDG VIRSAAVAIY
Consensus        ITC DS R      E       G G   VI   AGN   D        S   A

             ....|....| ....|....| ....|....| ....|....| ....|....|
                    110        120        130        140        150
AwCA         HLNTAVVLVM GHSHCG--AV AAAIEGHGEG YINAILEEIQ PVIVGETNVG
MtaCA        ALGVNEIIIV GHTDCGMARL DEDLIVSRMR ELGVEEEVIE NFSIDVLNPV
Consensus     L         GH  CG                        E I         N

             ....|....| ....|....| ....|....| ....|....| ....|....|
                    160        170        180        190        200
AwCA         KCENLNIENS IKRILQSEMI KTMIGEGQVK VLGCKYDLGS G---QVEFFE
MtaCA        GDEEENVIEG VKRLKSSPLI PESIG----- VHGLIIDINT GRLKPLYLDE
Consensus       E  N      KR   S  I    IG      V G   D    G        E

             ....|....

AwCA         NWSHPQFEK
MtaCA        DWSHPQFEK
Consensus     WSHPQFEK
```

Figure S15: ClustalW Multiple Alignment (BioEdit Sequence Aligment Editor v7.0.5) of β-CAs from *Methanobacterium thermoautotrophicum* (MtaCA) and *Acetobacterium woodii* (AwCA). Orange: StrepTag. Grey background highlights the zinc-coordinating histidines and blue background indicates amino acids which are involved in the catalytic reaction.

4. Codon Optimized CA Gene Sequences

AwCA-StrepTag

3`-CATATG

ATGATGAGCGAGAAGAAGGAGAACGTGATCAACGGTGTGATTAAGAAGCTGAAGCAAGGCAACGCGGACTTTGTG

AAGAACGAGAAAAACGAGGGCGACATCAGCCAGATGATTCGTACCAAGACCGTGGCGGAAGGTCAAAAACCGTAC

GCGGTGGTTATCACCTGCAGCGATAGCCGTGTTCCGCCGGAGCACATCTTCAGCGCGGGTATTGGCGAACTGTTT

GTGATCCGTACCGCGGGCAACGTTATTGGTGACTTCGAGCTGGGCAGCATCGAATACGCGGTGGGTCACCTGAAC

ACCGCGGTGGTTCTGGTGATGGGTCACAGCCATTGCGGTGCGGTTGCGGCGGCGATTGAGGGTCATGGTGAAGGT

TATATCAACGCGATTCTGGAGGAAATCCAGCCGGTGATTGTTGGCGAAACCAACGTTGGCAAGTGCGAGAACCTG

AACATCGAAAACAGCATCAAGCGTATTCTGCAGAGCGAGATGATCAAAACCATGATTGGCGAAGGTCAAGTGAAG

GTTCTGGGCTGCAAATATGATCTGGGTAGCGGTCAGGTTGAGTTCTTTGAAAACTGGAGCCATCCGCAATTTGAA

AAATAA

AAGCTT-5`

PmCA-StrepTag

3`-CATATG

GGCGGCGGTTGGAGCTACCACGGCGAGCATGGCCCGGAGCATTGGGGTGACCTGAAGGACGAGTACATTATGTGC

AAGATCGGCAAGAACCAGAGCCCGGTGGACATCAACCGTATTGTTGATGCGAAGCTGAAACCGATCAAGATTGAG

TACCGTGCGGGCGCGACCAAAGTGCTGAACAACGGTCACACCATCAAGGTTAGCTACGAACCGGGCAGCTATATC

GTGGTTGACGGTATTAAGTTCGAACTGAAACAGTTCCACTTTCACGCGCCGAGCGAGCACAAGCTGAAAGGCCAA

CACTATCCGTTCGAAGCGCACTTTGTGCACGCGGATAAACACGGTAACCTGGCGGTGATTGGCGTTTTCTTTAAG

GAAGGTCGTGAGAACCCGATCCTGGAAAAGATTTGGAAAGTGATGCCGGAGAACGCGGGCGAGGAAGTTAAGCTG

GCGCACAAAATCAACGCGGAGGACCTGCTGCCGAAAGACCGTGATTACTATCGTTATAGCGGCAGCCTGACCACC

CCGCCGTGCAGCGAGGGTGTGCGTTGGATTGTTATGGAGGAAGAGATGGAAATGAGCAAGGAACAAATCGAGAAG

TTTCGTAAAATTATGGGTGGCGATACCAACCGTCCGGTTCAACCGCTGAATGCGCGTATGATTATGGAGAAATGG

AGCCACCCGCAATTTGAGAAGTAA

AAGCTT-5`

MtaCA-StrepTag

3`-CATATG

CATCAAAGACATCCTGCGTGAGAACCAAGATTTCCGTTTCCGTGACCTGAGCGACCTGAAGCATAGCCCGAAGCT

GTGCATCATTACCTGCATGGACAGCCGTCTGATCGATCTGCTGGAACGTGCGCTGGGTATTGGCCGTGGTGACGC

GAAAGTGATTAAAAACGCGGGCAACATCGTGGACGATGGTGTTATTCGTAGCGCGGCGGTTGCGATCTATGCGCT

GGGCGTGAACGAGATCATTATCGTTGGCCACACCGATTGCGGTATGGCGCGTCTGGACGAGGATCTGATCGTGAG

CCGTATGCGTGAACTGGGTGTGGAGGAAGAGGTTATTGAGAACTTCAGCATCGACGTGCTGAACCCGGTTGGCGA

TGAAGAGGAAAACGTGATCGAAGGTGTTAAGCGTCTGAAAAGCAGCCCGCTGATTCCGGAGAGCATCGGCGTTCA

CGGTCTGATTATCGACATTAACACCGGTCGCCTGAAACCGCTGTATCTGGACGAAGACTGGAGCCATCCGCAATT

TGAGAAGTAA

AAGCTT-5`

StrepTag-SspCA

3`-CATATG

TGGAGCCACCCGCAATTTGAAAAGGAGCATGAGTGGAGCTACGAAGGCGAGAAAGGCCCGGAGCATTGGGCGCAA

CTGAAGCCGGAGTTTTTCTGGTGCAAACTGAAGAACCAGAGCCCGATCAACATTGACAAGAAATACAAGGTGAAA

GCGAACCTGCCGAAACTGAACCTGTACTATAAGACCGCGAAAGAGAGCGAAGTGGTTAACAACGGCCACACCATC

CAAATTAACATCAAAGAGGACAACACCCTGAACTACCTGGGTGAAAAATATCAGCTGAAGCAATTCCACTTTCAC

ACCCCGAGCGAGCACACCATTGAAAAGAAAAGCTATCCGCTGGAGATCCACTTCGTGCACAAAACCGAAGACGGC

AAGATTCTGGTGGTTGGCGTTATGGCGAAGCTGGGTAAAACCAACAAGGAGCTGGATAAAATTCTGAACGTGGCG

CCGGCGGAGGAAGGCGAAAAAATTCTGGACAAGAACCTGAACCTGAACAACCTGATCCCGAAGGATAAACGTTAC

ATGACCTATAGCGGCAGCCTGACCACCCCGCCGTGCACCGAGGGTGTGCGTTGGATTGTTCTGAAGAAACCGATT

AGCATCAGCAAGCAGCAACTGGAAAAGCTGAAAAGCGTGATGGTTAACCCGAACAACCGTCCGGTTCAGGAGATT

AATAGCCGTTGGATCATCGAAGGTTTCTAA

AAGCTT-5`

5. Calculated Hydratase Activities for Thermostability Experiments

Table S1. Calculated hydratase activities at 25 °C for AwCA and PmCA after preincubation for thermostability determination in turnover rate per mg enzyme and WAU per mg enzyme.

Temperature	Incubation Time	AwCA			PmCA		
		Total turnover rate µmol s^{-1} L^{-1}	Turnover rate per mg enzyme µmol s^{-1} mg^{-1}	WAU per mg enzyme	Total turnover rate µmol s^{-1} L^{-1}	Turnover rate per mg enzyme µmol s^{-1} mg^{-1}	WAU per mg enzyme
30 °C	96 h	1417 ± 37	2580 ± 75	1400 ± 165	1552 ± 59	3123 ± 120	2079 ± 327
	120 h	1408 ± 63	2349 ± 137	1368 ± 279	1530 ± 36	2837 ± 90	1956 ± 190
	144 h	1414 ± 40	2520 ± 99	1388 ± 185	1548 ± 32	3057 ± 86	2052 ± 175
40 °C	96 h	1289 ± 42	2068 ± 86	873 ± 160	1574 ± 11	3211 ± 26	2193 ± 58
	120 h	1222 ± 35	1606 ± 89	640 ± 118	1495 ± 41	2695 ± 99	1774 ± 208
	144 h	1199 ± 44	1662 ± 105	567 ± 136	1540 ± 8	3024 ± 60	2005 ± 44
50 °C	24 h	1262 ± 31	1944 ± 66	775 ± 111	1485 ± 25	2833 ± 55	1721 ± 124
	48 h	1090 ± 22	1115 ± 54	258 ± 53	1501 ± 31	2757 ± 64	1804 ± 156
	72 h	888 ± 28	489 ± 64	−105 ± 34	1480 ± 12	2859 ± 40	1700 ± 60
	96 h	868 ± 3	387 ± 17	−128 ± 3	1507 ± 8	2942 ± 23	1833 ± 42
	120 h	833 ± 26	47 ± 75	−159 ± 20	1474 ± 28	2610 ± 78	1667 ± 138
	144 h	813 ± 10	116 ± 61	−175 ± 6	1502 ± 9	2874 ± 60	1810 ± 45
60 °C	24 h	1116 ± 55	1204 ± 110	328 ± 138	1532 ± 28	2868 ± 59	1964 ± 149
	48 h	790 ± 24	−202 ± 64	−188 ± 12	1490 ± 12	2598 ± 49	1746 ± 62
	72 h	n. a.	n. a.	n. a.	1436 ± 9	2757 ± 20	1487 ± 41
	96 h	n. a.	n. a.	n. a.	1396 ± 2	2477 ± 35	1307 ± 8
	120 h	n. a.	n. a.	n. a.	1524 ± 14	2858 ± 37	1921 ± 71
	144 h	n. a.	n. a.	n. a.	1445 ± 49	2090 ± 105	1532 ± 231
70 °C	24 h	n. a.	n. a.	n. a.	1560 ± 33	3153 ± 68	2114 ± 182
	48 h	n. a.	n. a.	n. a.	1577 ± 17	3030 ± 38	2205 ± 96
	72 h	n. a.	n. a.	n. a.	1469 ± 10	2629 ± 24	1644 ± 51
	96 h	n. a.	n. a.	n. a.	1477 ± 36	2798 ± 79	1684 ± 176
	120 h	n. a.	n. a.	n. a.	1436 ± 10	2461 ± 57	1488 ± 45
	144 h	n. a.	n. a.	n. a.	1418 ± 26	2535 ± 77	1403 ± 117
80 °C	24 h	n. a.	n. a.	n. a.	1400 ± 27	2486 ± 61	1326 ± 119
	48 h	n. a.	n. a.	n. a.	1377 ± 33	2195 ± 69	1225 ± 140
	96 h	n. a.	n. a.	n. a.	1413 ± 29	2512 ± 59	1385 ± 130
	120 h	n. a.	n. a.	n. a.	1066 ± 18	979 ± 65	200 ± 41
	144 h	n. a.	n. a.	n. a.	1148 ± 37	1451 ± 79	412 ± 104
